# Lymphoid Neogenesis and Tertiary Lymphoid Organs in Transplanted Organs

**DOI:** 10.3389/fimmu.2016.00646

**Published:** 2016-12-27

**Authors:** Alice Koenig, Olivier Thaunat

**Affiliations:** ^1^Service de Transplantation, Néphrologie et Immunologie Clinique, Hôpital Edouard Herriot, Hospices Civils de Lyon, Lyon, France; ^2^INSERM UMR1111, Lyon, France; ^3^Université de Lyon, Lyon, France

**Keywords:** transplantation, lymphoid neogenesis, tertiary lymphoid organs, chronic rejection, tolerance

## Abstract

The progressive organization of immune effectors into functional ectopic lymphoid structures, named tertiary lymphoid organs (TLO), has been observed in many conditions in which target antigens fail to be eliminated by the immune system. Not surprisingly, TLO have been recurrently identified in chronically rejected allografts. Although significant progress has been made over the last decades in understanding the molecular mechanisms involved in TLO development (a process named lymphoid neogenesis), the role of intragraft TLO (if any) in chronic rejection remains elusive. The prevailing dogma is that TLO contribute to graft rejection by generating and propagating local humoral and cellular alloimmune responses. However, TLO have been recently observed in long-term accepting allografts, suggesting that they might also be able to regulate alloimmune responses. In this review, we discuss our current understanding of how TLO are induced and propose a unified model in which TLO can play deleterious or regulatory roles and therefore actively modulate the kinetics of chronic rejection.

## Introduction: The Challenge of Chronic Rejection in Transplantation

Vital organ failure is a life-threatening condition where a vital organ (i.e., kidney, heart, liver, or lung…) does not perform its expected function. Recent lifestyle changes in developed countries, and the increased incidence of chronic diseases such as hypertension, obesity, and diabetes, have set the stage for accelerated risk for, and the occurrence of, vital organ failure. As a result, vital organ failure is currently recognized as the leading cause of debility and premature death worldwide (www.who.int). In France alone, the personal, societal, and economic consequences of vital organ failure have a cost of more than €70 billion a year (25% of total health expenditures).

Transplantation consists in the restoration of vital physiologic functions through the surgical substitution of a defective organ by a functioning graft retrieved from a donor. Patients with end-stage vital organ failure depend on solid organ transplantation, which is their best (often their only) therapeutic option.

In clinical transplantation, the donor is from the same species but genetically different. Consequently, the immune system of the recipient inevitably recognizes the antigenic determinants (alloantigens) that differ between the recipient and the donor, particularly the highly polymorphic molecules from the major histocompatibility complex [i.e., human leukocyte antigen (HLA)] in humans. The alloimmune response that develops against the donor-specific HLA molecules is responsible for tissue damage, which leads to the failure of the transplanted organ, a process named “rejection.”

In the absence of a clinically applicable protocol able to induce the specific tolerance of the allogenic transplant by the recipient’s immune system (1, 2), the prevention of rejection is currently dependent upon immunosuppressive drugs (3). These drugs produce generalized immunosuppression, which means that any reduction in immune responsiveness to the allograft is accompanied by reduced immunity to infections and malignant diseases. Chronic immune injuries that result from the incomplete blockade of the recipient’s alloimmune response (i.e., chronic rejection) are currently the main factor limiting graft function duration (4). No significant progress has been made on this issue over the last decades as highlighted by the stagnation of graft half-life (5). A better understanding of the pathophysiology of chronic rejection is therefore a mandatory step in identifying innovative approaches that would prolong graft function duration.

## Intragraft Tertiary Lymphoid Organs (TLO)

Rejected grafts are characterized by interstitial infiltration of cellular effectors, mainly T cells and macrophages, but also dendritic cells, NK cells, B cells, and plasma cells.

In contrast with acute rejection, where infiltrates exhibit no particular spatial organization, during chronic rejection immune cells tend to organize themselves in structures that morphologically resemble the secondary lymphoid organs.

Analyzing all sorts of human kidney grafts removed for terminal chronic rejection, we and others showed that in the majority of chronically rejected grafts the immune cells were grouped, conferring a nodular organization to the infiltrate (6, 7). These nodules exhibited a highly organized microarchitecture with clear cell subset segregation: the core, made of the B cells intermingled with a network of follicular dendritic cells, was surrounded by T cells and mature dendritic cells. CD138-expressing plasma cells were found within or in close vicinity to TLO, suggesting that part of these cells differentiated locally. As in canonical secondary lymphoid organs the compartmentalization of the different cell subsets appeared to be mediated by gradients of homeostatic chemokines CCL21 (in the T cell area) and CXCL13 (in the B cell area). Furthermore, neolymphatic vessels and PNAd-expressing high endothelial venules (HEVs) were observed in the periphery of the nodules (8).

**Figure 1:**
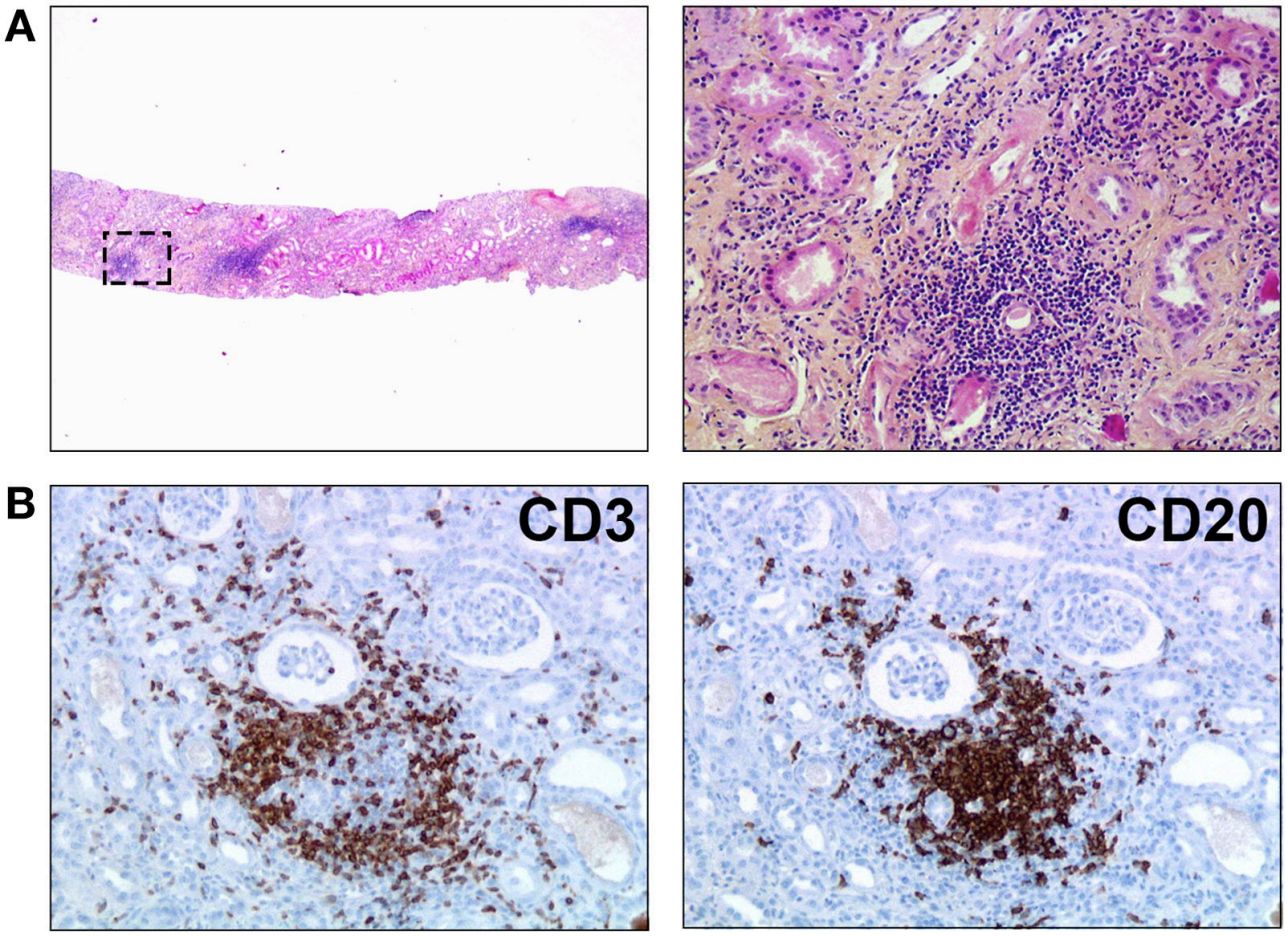
**Tertiary lymphoid organs in a chronically rejected renal transplant**. Biopsy of a renal transplant was performed for progressive deterioration of graft function, suggestive of chronic rejection. **(A)** HES staining revealed nodular infiltrates of mononuclear cells within graft parenchyma (original magnification: left panel, ×20; right panel, ×200). **(B)** Immunostainings unraveled the organized distribution of T cells (CD3+, left panel) and B cells (CD20, right panel). Original magnification: ×200.

The structural organization of immune effectors observed in chronically rejected renal grafts (Figure 1) does not seem specific of this type of transplant since similar lymphoid structures have been observed in chronically rejected pancreas, livers, hearts (7, 9–11), lungs (12), and even composite transplants (13–15). This phenomenon is not specific of the alloimmune setting either, since the very same lymphoid structures have previously been observed in various inflammatory conditions, including chronic infections, autoimmune diseases, and cancers (16, 17). Structural organization of immune effectors therefore appears as a generic response of the chronically stimulated immune system that cannot eradicate targeted antigens.

Because the microarchitecture of organized immune infiltrates is highly reminiscent of that of secondary lymphoid organs, these lymphoid structures have been named TLO.

## Molecular Mechanisms Involved in the Development of Secondary Lymphoid Organs

Primary immune responses are initiated in secondary lymphoid organs, which are located at strategic sites where antigens are most likely to be encountered.

The development of secondary lymphoid organs, a process named lymphoid organogenesis, is initiated during embryogenesis independently of antigen recognition at predetermined sites as a result of complex interactions between hematopoietic, mesenchymal, and endothelial cells (18, 19). Lymphoid organogenesis can be schematically divided into two consecutive steps: first the induction, then the organization phase.

The induction phase depends on lymphoid-tissue inducer cells, which arise in the fetal liver. Under the influence of TRANCE (at sites of peripheral lymph node development) or IL-7 (at mucosal sites) lymphoid-tissue inducer cells express membrane-bound lymphotoxin: a heterotrimer containing lymphotoxin α and lymphotoxin β that allow lymphoid-tissue inducer cells to interact with the lymphotoxin β receptor (LTβR) of stromal cells. Signaling through the LTβR initiates NFκB signaling in stromal cells, which promotes the production of homeostatic chemokines (18, 19).

Homeostatic chemokines are crucial for the organization phase. CXCL13 recruits circulating B cells to what becomes the B cell area of lymphoid tissues, and the T zone chemokines (CCL19 and CCL21) attract T and dendritic cells to shape the T cell area (18, 19). The lymphotoxin signaling pathway is also crucial in promoting the differentiation of HEVs, which are postcapillary venules expressing specific adhesion molecules (known as addressins) that have a crucial role in lymphocyte trafficking to secondary lymphoid organs (18, 19).

## Molecular Mechanisms of Lymphoid Neogenesis in Transplantation

**Figure 2:**
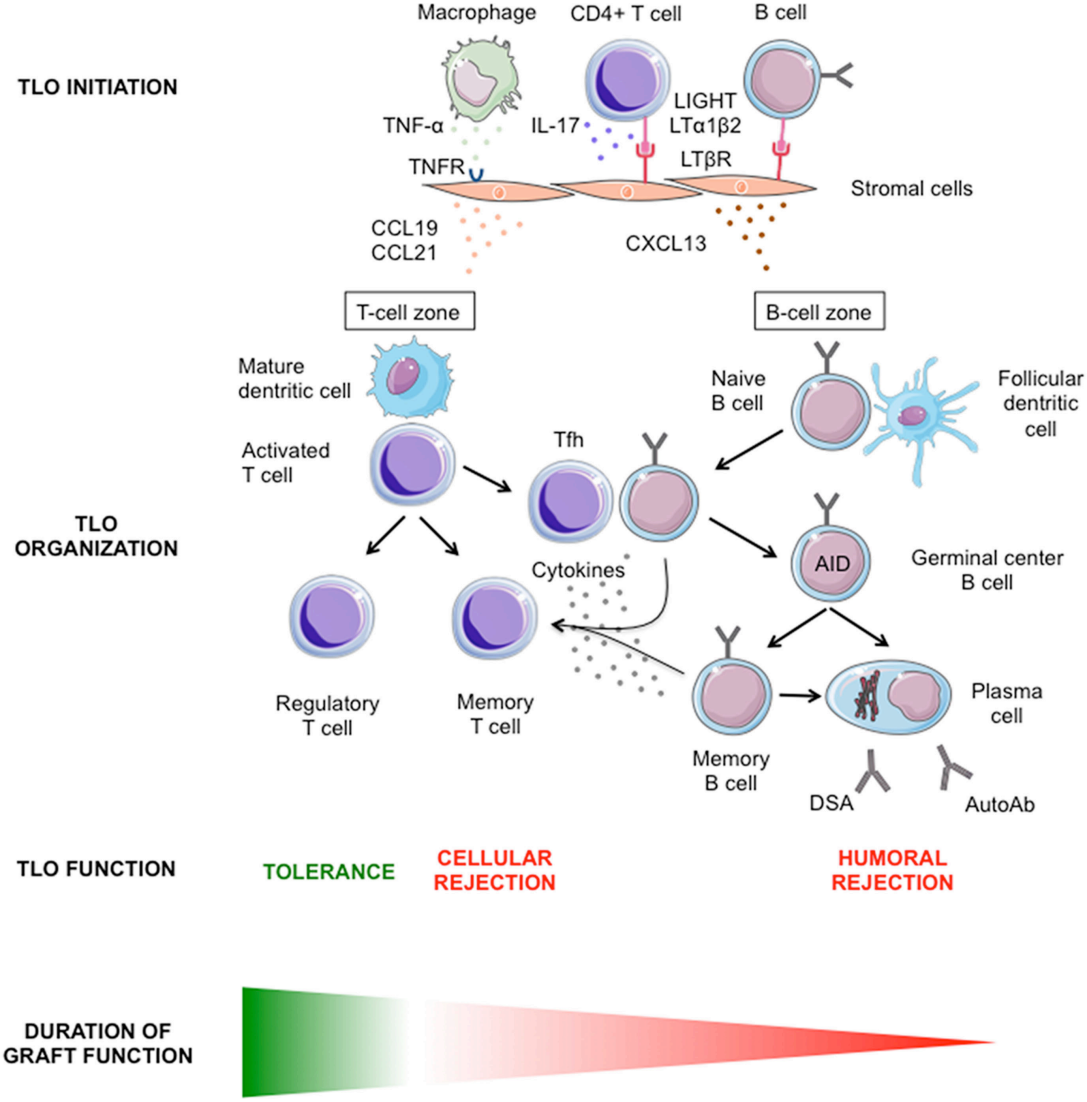
**Graphical summary of our current understanding of intragraft lymphoid neogenesis**. The main molecular mechanisms involved in the initiation (upper row) and the subsequent organization (middle row) of tertiary lymphoid organs (TLO) within transplanted organs are showed. The diverse functions of intragraft TLO and their respective impact on graft survival are presented (lower row).

Chronic rejection provides optimal conditions for studying the molecular mechanisms involved in the development of TLO (Figure 2). Indeed, (i) TLO have systematically been detected in chronically rejected grafts; (ii) the antigens targeted by the immune system are known (recipient-mismatched HLA antigens of the transplanted tissues); and (iii) chronically rejected grafts are sometimes removed, providing a large amount of diseased tissue, which can be comprehensively analyzed.

In-depth analysis of a series of detransplanted human renal grafts revealed the heterogenous nature of the cellular composition of TLO (8). Two types of B cell nodules could be identified: nodules composed of a uniform CD20^pos^ B cell population expressing IgD and Bcl-2 were similar to primary follicles, while nodules with a core of CD20^pos^IgD^neg^Bcl-2^neg^ B cells, highly expressing Bcl-6 that had pushed aside the CD20^pos^IgD^pos^Bcl-2^pos^ B cells, resembled secondary follicles, i.e., germinal centers (8). The ratio between these two types of structures differed between samples, and the number of ectopic germinal centers did not increase with the quantity of primary nodules. The phenotypic heterogeneity of TLO correlated more with the expression profile of a set of genes (*CCL19, CCL21, CXCL12, CXCL13*, CCR7, *CXCR4*, and *CXCR5*) involved in the formation and the maintenance of canonical secondary lymphoid organs (i.e., the lymphoid organogenesis described in the previous section) (18, 19). The complete recapitulation of this genetic program in chronically rejected grafts resulted in the generation of fully functional ectopic germinal centers that allowed for the efficient maturation of B cells into memory B cells and plasma cells (Figure 2). In contrast, when this recapitulation was incomplete, local B cell maturation was impeded (8). These results highlighted the similarity between the molecular processes involved in the development of canonical secondary lymphoid organs and those involved in the organization of immune effectors during chronic inflammation, a process named lymphoid neogenesis.

If the molecular mechanisms responsible for the organization and maintenance of secondary lymphoid organs and TLO appear similar, the initiation of the cascade is likely to be different (Figure 2). The formation of secondary lymphoid organs in the embryo is developmentally programed and results from the interaction between lymphotoxin-α_1_β_2_-expressing lymphoid-tissue inducer cells and lymphotoxin-β receptor-expressing stromal organizer cells (18, 19). In contrast, TLO development seems independent of lymphoid-tissue inducer cells (20, 21). Yet, several studies have documented the importance of the lymphotoxin pathway in lymphoid neogenesis (21, 22), including in a transplantation setting (23), by demonstrating that the development of TLO was abolished by treatment with inhibitory LTβR–Ig fusion protein. We must then ask who provides lymphotoxin signaling in the chronic rejection setting. Beyond lymphoid-tissue inducer cells, lymphotoxin-α and lymphotoxin-β are also expressed by activated lymphocytes (24). It is therefore conceivable that activated T and/or B cells replace lymphoid-tissue inducer cells to initiate lymphoid neogenesis in rejected grafts (Figure 2) as already demonstrated for the induction of TLO in the gut (21). Another possibility is that lymphotoxin is dispensable for the formation of TLO. Lymphotoxin-α and lymphotoxin-β are two related members of the large TNF ligand family (25). Since homologous genes and gene products often have redundant physiological functions, it seems reasonable to propose that other ligands and/or receptors of the TNF superfamily could act as alternative pathways for TLO induction (Figure 2). In line with this hypothesis, the provision of the alternative LTβR ligand LIGHT (aka tumor necrosis factor superfamily member 14) by activated T cells infiltrating inflamed pancreas have been shown to be crucial for the formation of TLO (26). Furthermore, TNF-α, which is produced within rejected grafts (27), has been shown to be critical for the development of TLO in a murine model of atherosclerosis (20). TNF-α does not bind to LTβR but to distinct TNF receptors (25). Using apolipoprotein E-deficient mice, which spontaneously develop atherosclerotic lesions in their aorta, the Antonino Nicoletti’s group recently demonstrated that the blockade of LTβR signaling had no effect, whereas that of TNFR1/2 signaling reduced the expression of homeostatic chemokines and the subsequent development of TLO (20). Finally, it has recently been reported that IL-17 produced by CD4+ T cells (i.e., Th17 cells) was essential for the formation of both (i) TLO in the central nervous system of mice during experimental autoimmune encephalomyelitis (the animal model of multiple sclerosis) (28) and (ii) the development of inducible bronchus-associated lymphoid tissue, an ectopic lymphoid tissue that forms in the lungs after pulmonary inflammation (29, 30). In the latter setting, IL-17 acted by triggering the expression of homeostatic chemokines independently of lymphotoxin signaling (Figure 2). If this hypothesis was proven true in transplantation, initiation of lymphoid neogenesis in chronically rejected grafts could therefore be totally independent of both lymphoid-tissue inducer cells and the lymphotoxin/TNF pathway. Interestingly, we have recently reported that a Th17 polarization of CD4+ T cells infiltrating the graft was associated with increased TLO development during clinical chronic rejection (31).

It is conceivable that instead of conflicting with each other, these different works reveal the fact that several pathways can promote the initiation of TLO depending on the initiating events. This hypothesis was recently substantiated by the demonstration that the development of bronchus-associated lymphoid tissue was triggered by different pathways according to the pathogen responsible for lung inflammation (29).

While significant progress has been made in the identification of the molecular mechanisms that participate to the development of TLO, the endogenous signals capable of inhibiting the lymphoid neogenesis are far more elusive. Through evaluation of synovial tissues from rheumatoid arthritis patients it has been recently reported that low interleukin-27 (IL-27) expression corresponds with an increased incidence of TLO and gene signatures associated with their development and activity. The presence of synovial TLO was also noted in mice deficient in the IL-27 receptor after the onset of inflammatory arthritis (32). IL-27 might therefore represent a negative regulator of TLO development. Whether this is also true for chronic rejection remains to be demonstrated.

## Do Intragraft TLO Promote Chronic Rejection?

Tertiary lymphoid organs differ from canonical secondary lymphoid organs inasmuch as they develop in an inflammatory milieu (31, 33), enriched in neoantigens released from injured tissue and trapped by defective lymphatic drainage (34). Comparing the cellular composition of TLO of chronically rejected grafts with one of the secondary lymphoid organs, we observed a drastic increase in the percentage of activated and memory CD4+ T cell in intragraft TLO and a symmetric decrease in T regulatory subsets (IL-10-producing Tr1 cells and Foxp3^pos^ Tregs) in both a murine experimental model and human samples (33, 35).

These peculiarities suggest that the local immune response that develops in intragraft TLO might be less tightly regulated than in secondary lymphoid organs and are therefore more aggressive. In line with this hypothesis, we (33) and others (23) have shown that intragraft TLO are a major site where B cell tolerance breakdown occurs during chronic rejection (Figure 2). Interestingly, the generation of autoantibodies following solid organ transplantation has long been reported to correlate with chronic rejection, and the deleterious impact of some autoantibodies on graft survival has been demonstrated (36, 37). Furthermore, comparing the alloimmune responses elicited in intragraft TLO, spleen, and draining lymph nodes in a rat model of chronic rejection, our group observed increased production of anti-HLA antibodies in TLO as compared with canonical secondary lymphoid organs (35). Not only were the humoral alloimmune responses elicited in TLO quantitatively enhanced but they also displayed a more diverse repertoire, a finding that we confirmed in the clinical setting by the analysis of chronically rejected human kidney allografts (8).

Tertiary lymphoid organs could also contribute to chronic destruction of the graft through antibody-independent functions of B cells. B cells are indeed unique antigen-presenting cells because (i) they have an antigen-specific receptor (B cell receptor), which when engaged by surface-tethered antigens leads to the formation of an immunological synapse that coordinates cell signaling events and promotes antigen uptake for presentation on MHC class II molecules (38), even when the antigen is membrane-tethered or is present in limiting quantities and (ii) B cells have the capacity to clonally expand, thereby becoming the numerically dominant antigen-presenting cells. Interestingly, it has been reported that the presence of B cell clusters within the graft during rejection was associated with reduced graft survival and resistance to steroid therapy, independently of C4d (a breakdown product generated during classical complement pathway activation) deposition or alloantibody detection (39). Some authors have proposed that this could be due to the local presentation of antigen to effector T cells by intragraft B cells (40). This hypothesis is supported by experimental data from the group of Fadi Lakkis, who showed that in a murine skin graft model, TLO perpetuate the rejection process by supporting naïve T cell activation within the graft (41). Strikingly, the same authors also demonstrated that TLO generate T cell memory immune responses (41).

In addition to presenting antigen, B cells can also enhance T cell-mediated immune responses through the secretion of cytokines and chemokines. Studies from the group of Frances Lund (42) have shown that B cells can be functionally subdivided based on their cytokine profile. B cells activated in the presence of TH1-type cytokines (referred to as Be-1 cells) secrete IFNγ and IL-12 but not IL-4, IL-13, or IL-2. By contrast, B cells activated in the presence of TH2-type cytokines (Be-2 cells) secrete IL-2, lymphotoxin, IL-4, and IL-13 but make minimal amounts of IFNγ and IL-12. Both Be-1 and Be-2 cells seem able to produce IL-10, TNFα, and IL-6. The importance of B cell cytokines in promoting T cell responses has been illustrated in several models. For example, *in vitro* generated effector B cells that produced either TH1- or TH2-type cytokines were shown to promote the activation and differentiation of naïve T cells into effector TH1 and TH2 cells, respectively (43). The importance of B cell cytokines in promoting T cell responses has been confirmed *in vivo*. In a murine model of *Toxoplasma gondii* infection, TNF production by B cells was shown to be required for the generation of an optimal TH1 cell protective response (44). In another set of experiments, the generation of a protective TH2 memory response to *H. polygyrus* was shown to depend on IL-2-producing B cells (45). The exact role of cytokine-producing B cells in enhancing intra-TLO T cell responses remains to be evaluated.

Since grafts in which TLO were harboring germinal center reactions had a shorter life expectancy (Figure 2), we have proposed that lymphoid neogenesis could play a detrimental role during chronic rejection (8). However, the validity of this conclusion is limited by the fact that only explanted grafts have been analyzed, i.e., organs displaying extreme rejection damage that are sometimes (notably in the case of renal grafts) removed after immunosuppressive therapy withdrawal. The definitive demonstration that TLO are involved in the pathophysiology of chronic rejection would require selectively impairing the development of intragraft TLO while leaving the rest of the recipient’s immune system unaffected. Addressing this issue is not trivial because, as discussed above, TLO share many biological pathways with canonical lymphoid tissue, and hence an adequate experimental model is not currently available. Therefore, most of the attempts to validate the data obtained in murine experimental models and in human detransplanted grafts have relied on graft biopsies. The identification of TLO within the grafts before the development of the lesions indeed appears as a prerequisite for confirming the role of lymphoid neogenesis in chronic rejection. This implies a study of protocol biopsies, which has long been introduced as standard follow up in transplantation (46). Unfortunately, the numerous studies aiming at evaluating the correlation between the presence of TLO in protocol biopsies and the later development of chronic rejection have reached conflicting conclusions (Table 1).

**Table 1:** **Summary of biopsy-based studies evaluating the role of graft-infiltrating B cells**.

Reference	Population	Biopsy indication	Histologic criteria	Key findings
**KIDNEY RECIPIENTS**				
Sarwal et al. (39)	51 patients	Biopsy with acute graft rejection	CD20+ cell count >275/HPF	B cell clusters associated with glucocorticoid resistance and graft loss
Hippen et al. (58)	27 patients	Biopsy with Banff 1A or 1B acute rejection	CD20+ if “strong and diffuse staining”	CD20+ correlated with steroid-resistance rejection and reduced graft survival
Kayler et al. (59)	120 patients	Biopsy with first episode of acute cellular rejection	Cluster of ≥15 CD20+ cells in the tubulo-interstitial compartment	CD20+ clusters are not prognostic factors for glucocorticoid resistance and graft loss
Bagnasco et al. (60)	58 patients (74 biopsies)	Biopsy with type 1 and type 2 acute cellular rejection during the first year post-Tx	B cell-rich when ≥1 cluster containing 100 CD20+ cells/HPF	No correlation between B cell-rich biopsies and worst graft outcome
Scheepstra et al. (61)	50 patients (54 biopsies)	Biopsy with clinically suspect and histologically confirmed acute rejection	B cell (CD20+) count >275/HPF CD20+ cluster if >30 cells CD20+ without the interposition of tubules	Presence of B cells does not correlate with response to conventional therapy or graft outcome
Hwang et al. (62)	54 patients (67 biopsies)	Biopsy with acute cellular rejection	CD20+ count >275/HPF CD38+ if >30% infiltration	CD38+ B cells ± CD20+ B cells correlated with poor clinical outcomes
Martin et al. (63)	18 patients	Serial biopsies for 10 recipients with chronic dysfunction and 8 with long-term normal graft function	Plasma cells count Cd4 deposits DSA elution from biopsy	Patients developing chronic rejection present plasma cells, DSA, and C4d depositions more often than control group on their biopsy
Abbas et al. (64)	50 patients	Biopsy for cause	Plasma cell-rich acute rejection if >10% plasma cells	Plasma cell-rich acute rejection correlated with a poor graft outcome when associated with DSA
**HEART RECIPIENTS**				
Yamani et al. (65)	140 patients	Systematic biopsy	Nodular endocardial infiltrates (quilty lesions)	Quilty lesions are associated with increased development of coronary vasculopathy at 1 year
Chu et al. (66)	285 patients	Systematic biopsy	Quilty lesions	Patients with quilty lesions and no anti-HLA class II DSA are more likely to develop graft arteriosclerosis at 5 years
Hiemann et al. (67)	873 patients (9,713 biopsies)	Systematic biopsy	Quilty lesions	Quilty lesions are associated with an increased risk for stenotic microvasculopathy and a poor graft outcome
Zakliczynski et al. (68)	344 patients	Systematic biopsy	Quilty lesions	Positive correlation between quilty lesions and an increased risk of acute rejection but not with the occurrence of coronary artery vasculopathy
Frank et al. (69)	79 patients (37 with DSA)	Biopsy with or without graft dysfunction	Ratios of T:B cells and CD4:CD8 T cells	Patients with DSA have lower CD4:CD8 T cell ratio than controls T:B cell ratio was similar in patients with and without DSA
**COMPOSITE TISSUE RECIPIENTS**				
Hautz et al. (14)	6 human hand recipients (187 biopsies)	Systematic and for cause biopsies	CD3, CD4, CD8, CD20 PNAd stainings	PNAd expression in graft vessels correlated with rejection and T- and B-cell infiltration

*DSA, donor-specific antibodies; HLA, human leukocyte antigen; HPF, high power field; PNAd, peripheral lymph node addressin; Tx, transplantation*.

The absence of an unequivocal deleterious role for B cell clusters has led to the conclusion that these structures could be like “fish in a sunken ship,” i.e., although fish are frequently seen in a sunken boat, they play no role in the process responsible for the shipwreck.

## Intragraft TLO: Friends and Foes?

An alternative explanation could reconcile these apparently conflicting results. As discussed above, the proportion of B cells that infiltrate chronically rejected kidney grafts does not correlate with the functionality of intragraft TLO (8). The attraction of B cells within inflamed tissue appears therefore to be a generic phenomenon with no intrinsic deleterious consequences on the graft. However, when intragraft B cells meet the appropriate microenvironment, and upon the complete recapitulation of the lymphoid organogenesis program, B cell nodules organize themselves into functional ectopic germinal centers, which harbor the development of a local aggressive immune response. Because graft biopsies provide only a very limited amount of tissue (which is already an important limitation for evaluation in a patchy process such as lymphoid neogenesis), they do not allow for functional analysis of the ectopic lymphoid organs and are therefore inappropriate for analyzing the role of B cell clusters in rejected grafts.

Another layer of complexity has recently been brought into the picture by experimental evidence that certain B cell subsets are endowed with an immune regulatory role (47). For instance, IL-10-producing B cells have been shown to efficiently prevent the induction of autoimmune disease in several mouse models (48–50). Tolerance in transplantation is defined as the maintenance of graft function in the absence of therapeutic immunosuppression for at least 12 months. About 100 tolerant patients have been identified among renal transplant recipients over the last decade (51). These patients, defined as “operationally tolerant,” are healthy, do not exhibit more infections or malignancies than healthy volunteers, and do not display clinical evidence of immune incompetence (51). When compared with transplanted patients with stable graft function under pharmacologic immunosuppression, operationally tolerant patients exhibited an increase in both absolute number and frequency of total B cells (52). Furthermore, two independent microarray analyses of PBMC revealed a higher expression of B cell-related genes and their associated molecular pathways in tolerant recipients (53, 54). It is therefore conceivable that in certain conditions intragraft B cell infiltrate, instead of being neutral or deleterious, could actually promote graft survival (Figure 2). This theory has been nicely illustrated by murine experimental studies that recently reported the formation of TLO within tolerated allografts (55–57). If such a local protective response can prevent terminal failure of grafts, then not only would such samples having “tolerogenic” TLO be absent from the studies based on the analysis of detransplanted grafts but it could also explain the difficulty of biopsy-based studies to reach an unequivocal conclusion.

## Conclusion

Transplanted organ expresses donor-specific alloantigens, which stimulate a recipient’s immune system. Prevention of acute rejection of the graft is achieved using a combination of non-specific immunosuppressive drugs that can only partially block the alloimmune effectors. The residual enduring alloimmune response promotes immune injuries known as chronic rejection, the main cause of late allograft loss. As in other chronic immune diseases, immune effectors within chronically rejected allografts progressively organize into functional TLO that display the same microarchitecture as secondary lymphoid organs, a process known as lymphoid neogenesis. Because biopsy-based studies have reached conflicting conclusions regarding the pathological significance of these TLO, it has been proposed that the presence of TLO in rejected grafts is a non-specific response to local inflammation-induced production of chemokines. While that can indeed sometimes be the case, it should not be excluded that under appropriate conditions, lymphoid neogenesis turns non-functional TLO into ectopic germinal centers, in which a local aggressive humoral immune response can be elicited. Alternatively, functional TLO can also regulate immune responses and slow down the destruction process.

Therefore, we propose that TLO be considered as active players, able to modulate the kinetics of the natural history of chronic rejection. Future works will determine if the versatility of TLO can be manipulated to design innovative therapeutic interventions that would improve graft life expectancy.

## Author Contributions

All the authors listed have made substantial, direct, and intellectual contribution to the work and approved it for publication.

## Conflict of Interest Statement

The authors declare that the research was conducted in the absence of any commercial or financial relationships that could be construed as a potential conflict of interest.
